# Obesity and Breast Cancer: Role of Leptin

**DOI:** 10.3389/fonc.2019.00596

**Published:** 2019-07-18

**Authors:** Flora Sánchez-Jiménez, Antonio Pérez-Pérez, Luis de la Cruz-Merino, Víctor Sánchez-Margalet

**Affiliations:** ^1^Department of Medical Biochemistry and Molecular Biology, and Immunology, School of Medicine, Virgen Macarena University Hospital, University of Seville, Seville, Spain; ^2^Department of Clinical Oncology, Virgen Macarena University Hospital, University of Seville, Seville, Spain

**Keywords:** leptin signaling, leptin receptor, breast cancer, obesity, leptin

## Abstract

Obesity-related breast cancer is an important threat that affects especially post-menopausal women. The link between obesity and breast cancer seems to be relying on the microenvironment generated at adipose tissue level, which includes inflammatory cytokines. In addition, its association with systemic endocrine changes, including hyperinsulinemia, increased estrogens levels, and hyperleptinemia may be key factors for tumor development. These factors may promote tumor initiation, tumor primary growth, tissue invasion, and metastatic progression. Although the relationship between obesity and breast cancer is already established, the different pathophysiological mechanisms involved are not clear. Obesity-related insulin resistance is a well-known risk factor for breast cancer development in post-menopausal women. However, the role of inflammation and other adipokines, especially leptin, is less studied. Leptin, like insulin, appears to be a growth factor for breast cancer cells. There exists a link between leptin and metabolism of estrogens and between leptin and other factors in a more complex network. As a result, obesity-associated hyperleptinemia has been suggested as an important mediator in the pathophysiology of breast cancer. On the other hand, recent data on the paradoxical effect of obesity on cancer immunotherapy efficacy has brought some controversy, since the proinflammatory effect of leptin may help the effect of immune checkpoint inhibitors. Therefore, a better knowledge of the molecular mechanisms that mediate leptin action may be helpful to understand the underlying processes which link obesity to breast cancer in post-menopausal women, as well as the possible role of leptin in the response to immunotherapy in obese patients.

In western countries, the incidence of breast cancer has increased more than 30% in the last 25 years. Even though this is attributed in part to changes in reproductive patterns and improved detection methods, this increase may also reflect the increasing prevalence of obesity and physical inactivity in the population (1).

Overweight and obesity seem to be inversely related to the incidence of breast cancer in pre-menopausal women. However, in the development of post-menopausal breast cancer, as happens with other tumors, obesity has been considered a major risk factor. A meta-analysis of prospective observational studies revealed this relationship, concluding that there is an increased risk of 12% of breast cancer in post-menopausal women for each increase of 5 kg/m^2^ in body mass index (BMI) (2).

Increased adiposity has been correlated with large tumor size, tumor node positivity and poor prognosis of breast cancer (3). Obesity affects many hallmarks of cancer, including genomic instability, tissue metabolic changes, altered extracellular matrix properties, dysregulation of cell death, immune system, and angiogenesis (4).

The aim of this article is to review the current literature to better understand the relationship between obesity and breast cancer and to compile the known molecular mechanisms underlying this association. Finally, we will summarize new data regarding the obesity paradox based on the beneficial effect of overweight on the results of immunotherapy of cancer and discuss the possible role of leptin.

## Main Mechanisms Involved in Obese Associated Breast Cancer

**Figure 1:**
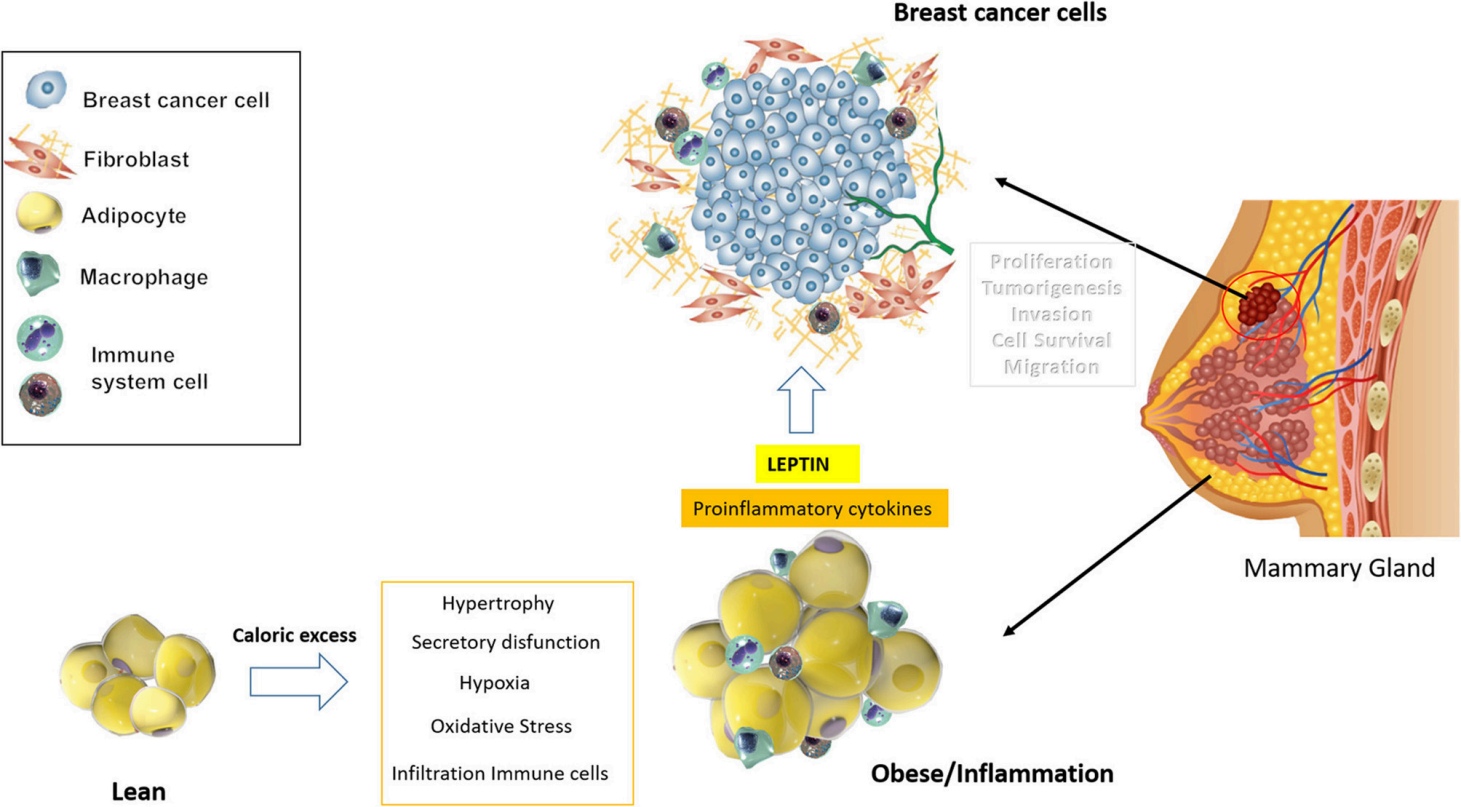
Breast cancer and obesity. The picture summarizes the mechanisms that connect obesity or excess of adipose tissue with the development of breast cancer.

Several mechanisms closely related to each other have been proposed to explain specifically the association between breast cancer and obesity, including chronic subclinical inflammation, sex hormone deregulation, insulin/IGF-1 pathways, and the secretion of different adipokines, where leptin plays a key role (Figure 1).

### Chronic Inflammation

It has been demonstrated that adipose chronic inflammation increases the survival and proliferation of cancer cells, promotes angiogenesis, metastatic spread, alters antitumor immune responses, and interfere with hormonal or chemotherapeutic treatments having a role in cancer prognosis (5, 6). At the tumor site, the presence of histological focal inflammatory points known as crown-like structures (CLS) and the proximity of adipose stromal cells, lead to the generation of a proinflammatory microenvironment. Thus, there are increased levels of infiltrated M1 macrophages, cytokines and pro-inflammatory mediators, such as cyclooxygenase-2 (COX-2), TNF-α, IL-6, monocyte chemoattractant protein-1 (MCP-1), and interleukin (IL)-1β (7) around the tumor site. Several inflammatory mediators produced by inflammatory cells also regulate leptin expression in this context and promote the development of chronic inflammation (8). The overall leptin action in the immune system is to activate proinflammatory cells, promote T-helper 1 responses, and mediate the production of the other proinflammatory cytokines (9, 10). Inflamed adipose tissue also induces neutrophil differentiation, leading to increased neutrophilia that seems to be mediated by high levels of GM-CSF. This is also responsible for the recruitment of several immunosuppressive cells in the adipose microenvironment, namely T-regulatory (T-reg) cells, myeloid-derived suppressor cells (MDSCs), and tumor-associated macrophages (TAMs) (11, 12).

### Sex Hormone Deregulation

After menopause, adipose tissue becomes the main producer of estrogens through the enzymatic conversion of androstenedione to estrone, carried out by aromatase from stromal cells. In obese women, aromatase activity is elevated resulting in increased estrogen production and in relatively high plasma levels. This is associated with an increase in the risk of breast cancer and with the worst prognosis observed in this population (13, 14).

Factors produced during inflammation and the paracrine loop that is established between macrophages and adipose stromal cells may induce the aromatase enzyme, with the consequent stimulation of ER-positive breast cancer epithelial cell growth through locally produced estrogens. Thus, there seems to be a strong correlation among obesity, inflammation, and hormone-receptor positive cancer (15, 16). This association is in line with the major role that estrogens synthesized by stromal adipose tissue have in the pathogenesis of these tumors.

Other factors that are related to the deregulation of sex hormones in this context are the adipose tissue containing 17β-hydroxysteroid dehydrogenase, which is responsible for the more biologically potent conversion of estrone to estradiol, as well as the decrease of the sex hormone binding globulin (SHBG) levels, which in turn, increases the level of biologically available estradiol (13).

### Insulin

As part of the adipose metabolic alterations, insulin is another obesity-related factor that induces the activity of adipose aromatase, while directly stimulating the growth and invasion of breast cancer cells (16–18). The coexistence of deregulated insulin signaling in obesity has been well-known for decades. BMI correlates directly with circulating insulin and insulin-like growth factor-1 (IGF-1). High levels of insulin, as a consequence of insulin resistance in obese women, are also associated with an increased risk of post-menopausal breast cancer as well as an increased risk of cancer recurrence and mortality (1, 13). Hyperinsulinemia contributes to the development of cancer due to the direct effects exerted by insulin promoting growth, and indirectly, due to the decrease of circulating levels of IGF-1 binding proteins (IGFBP3), which increases the bioavailability of this growth factor. High level of IGF-1 has been associated with increased risk of breast cancer in both pre-menopausal and post-menopausal women (19). Also hyperglycemia seems to increase the risk of breast cancer in pre-malignant cells and to enhance cancer progression in malignant epithelial cells through leptin/IGF1 signaling (20).

The binding of insulin or IGF-1 to its specific receptors activates several growth promoting signaling pathways, including MAPK and PI3K/Akt/mTOR. Additionally, insulin prevents apoptosis by inducing increased expression of the anti-apoptotic proteins Bcl-2 and Bcl-XL, and the concomitant suppression of the pro-apoptotic protein Bax. Increased signaling by insulin, positively regulates vascular endothelial growth factor (VEGF) and the hypoxia-inducible factor 1α (HIF-1α) to promote tumor angiogenesis, proliferation of endothelial cells, and the formation of blood vessels. All of them, mechanisms involved in the development, progression, and invasion of breast cancer (21, 22).

### Adipokines

Aberrant adipokine and cytokine signaling represents one of the key features of obese metabolism and the physiopathology of obesity-related diseases. Adipocytes in obese individuals produce endocrine, inflammatory and angiogenic factors to affect adjacent breast cancer cells. Traditionally, the two most important adipokines associated with the development of breast cancer related to obesity are leptin, which will be extensively reviewed below, and adiponectin (23). Adiponectin exerts a negative regulatory function in obesity-related breast carcinogenesis. Hence, low levels of adiponectin, characteristic of obesity, are associated with increased proliferative activity, resulting in an increased risk of developing cancer. In addition, low serum adiponectin levels are also associated with a larger tumor size and a poorer prognosis of breast cancer (24). Based on the antagonistic impact on the risk of breast cancer, some authors have proposed that the adiponectin/leptin ratio may be more useful than the individual levels of adipokines for assessing the risk profile of breast cancer (25). Adiponectin increases the expression and activity of the physiological inhibitor of leptin signaling protein tyrosine phosphatase 1B (PTP1B) (26) and activates adenosine monophosphate-activated protein kinase (AMPK), which has a variety of cellular effects including induction of cell cycle arrest and inhibition of mammalian target of rapamycin (mTOR) activity. More recently, adiponectin has been related to the induction of autophagic cell death in breast cancer (27).

## Leptin and Breast Cancer

One of the pathophysiological factors of breast cancer in obesity is hyperleptinemia. Leptin is considered both hormone and adipokine and it is produced and secreted into circulation mainly by adipose tissue. It is also synthesized and secreted by placenta and in small amounts, by the mucosa of the gastric fundus, skeletal muscle, brain, bone marrow, lymphoid tissues, immune cells, ovaries, endometrium during embryo implantation, and both normal and malignant tissue of the breast (28, 29).

Both leptin secretion and leptin circulation present fluctuations that follow a circadian rhythm and they also change with nutritional status. Circulating levels of leptin communicate the state of energy storage to the brain. These levels reflect the amount of existing adipose tissue, increasing in proportion to the BMI. In addition, it has been demonstrated that serum leptin levels are higher in women compared to men, even after correction of body weight. This could be explained by subcutaneous synthesis and estrogens and androgens regulation (30).

The main function of leptin is the maintenance of energy homeostasis, participating in the anorexigenic pathway through a central feedback mechanism at the level of the hypothalamus. In this way, it controls the growth of adipose tissue through intermediate hormonal mechanisms that regulate food intake. In addition to this main function, it is known that leptin has effects on fetal development, reproduction, lactation, bone development, hematopoiesis, immune response, angiogenesis, and the proliferation of many different cell types, including cells of breast tissue (25, 29, 31). One of the peripheral functions of leptin is a regulatory role in the interplay between energy metabolism and the immune system, in part responsible for the inflammatory state associated to obesity (32).

Elevated leptin levels has been linked to breast cancer aggressiveness and bad prognosis in epidemiological studies (33, 34). Moreover, this hormone/cytokine has been recently suggested as a biomarker that could be associated with type, grade, stage, lymph node involvement, hormone receptors, and recurrence in breast cancer based on its immunohistochemical staining (35).

Leptin signaling has been shown to regulate various important molecules involved in proliferation, adhesion, invasion, migration, inflammation, and angiogenesis, as it has been demonstrated in breast carcinogenesis with the regulation of the expression of cyclin D1, p53, survivin, IL1, E-Cadherin, vascular endothelial growth factor and its receptor type 2, and several tissue factors (36–44).

**Figure 2:**
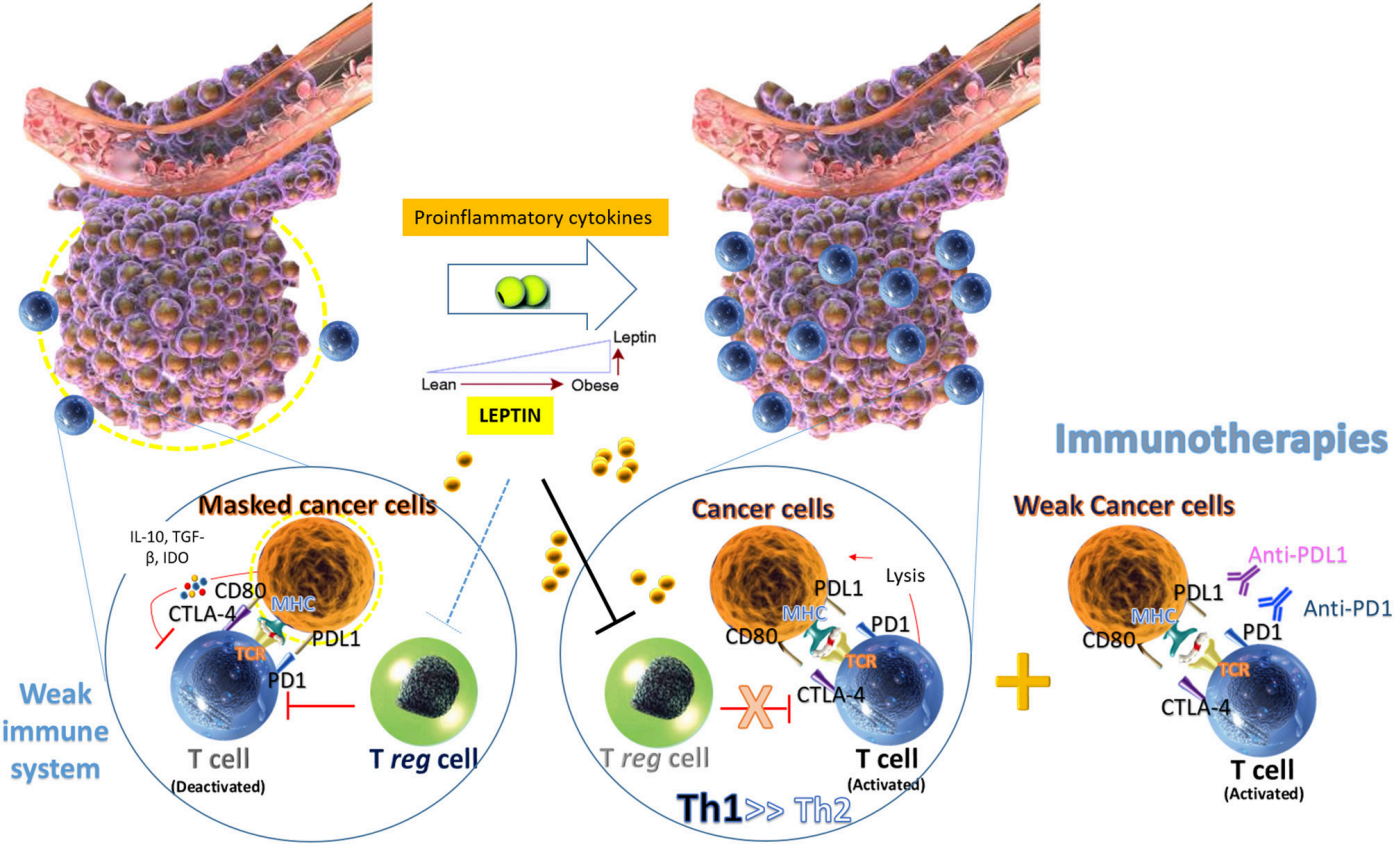
Beneficial effect of obesity on the effects of immunotherapy of cancer. Obesity is commonly associated with an “inflammatory” state with increased pro-inflammatory immune cells as well as increased levels of inflammatory cytokines, such as leptin. In this environment, leptin could promote the switch toward T helper 1 (Th1) cell immune responses and could act as a negative signal for the expansion of human regulatory T cells (Treg). Low BMI may be useful for the immune escape, whereas high BMI and obesity, via inflammatory adipokines, such as leptin may help to the immunotherapy of cancer, providing support to the increased immune response of the host to the tumor.

In breast cancer, leptin has been proposed to have a role at different levels (45, 46). At a carcinogenic level, it has been suggested to act as a direct potential activator of short term ROS production in human epithelial mammary cells (47), although other studies previously showed that chronic leptin treatment decreases ROS levels and oxidative stress in MCF-7 cells (48). According to the trophic function of leptin, demonstrated also in MCF7 cells (49), it seems to regulate metabolic reprogramming to promote cellular growth (50). However, the pro-carcinogenic effect of leptin in breast cancer results not only from an enhanced activity of signaling pathways involved in the proliferation process, but also from a probable down-regulation of the apoptotic response (51). Thus, a cardinal role of leptin in autophagy and suppression of apoptosis in cancer cells has been proposed as well (52). Additionally, leptin has been considered as a mediator of tumor-stromal interactions (53), where it seems to participate in the crosstalk between breast cancer cells and tumor-associated macrophages M2 by stimulating IL-18 and IL-8 production to promote tumor growth and metastasis (54) (Figure 2). More recently, leptin has been proposed to promote cancer stem cells (CSC) enrichment and epithelial-to-mesenchymal transition (EMT) phenotype by upregulating the expression of multiple CSC/EMT–related genes (55).

Regarding regulation of leptin expression in breast carcinogenesis, it is remarkable that hyperinsulinemia induces breast cancer progression also through leptin-expression-dependent mechanisms (56). One of the mechanisms of leptin overexpression in breast tumors seems to involve HIF-1α, a component of HIF transcriptional factor that can be upregulated by hypoxia and hyperinsulinemia (57). Moreover, a recent study has demonstrated that obesity reduces the level of the tumor suppressor p16^INK4A^ protein in breast adipocytes, which in turn seems to upregulate leptin expression at mRNA level to promote also procarcinogenic processes (58). On the other hand, other studies have suggested a role for the protein BMP9 as a negative regulator of leptin expression in breast cancer cells (59).

### Leptin Receptor

The mechanism of leptin action starts with the activation of its transmembrane receptor, called Ob-R or LEPR, which has a helical structure and is related to class I cytokine receptors. LEPR is present in tissues such as pancreas, placenta, adrenal glands, stomach, hematopoietic cells, liver, heart, lung as well as breast cells (30).

These receptors lack autophosphorylation capabilities so they need auxiliary kinases for their activation. The Ob-R/LEPR receptor has 6 isoforms resulting from different alternative splicing of the gene: there are four of them with short cytoplasmic domains (Ob-Ra, Ob-Rc, Ob-Rd, and Ob-Rf), a long form Ob-Rb and a soluble form Ob-Re, whose main function is to control serum leptin levels. These six isoforms share an extracellular domain of common leptin binding capacity, but they differ in their intracellular domains. Ob-Ra, Ob-Rb, Ob-Rc, Ob-Rd, and Ob-Rf receptors are transmembrane receptors that contain the domain called Box1, required for the binding of janus kinase 2 (JAK2), to activate the PI3K and MAPK signaling pathways. However, only Ob-Rb has a coupling site (box 2) essential for the activation of the JAK-STAT pathway and it is the only leptin receptor that has a potentially phosphorylated intracellular domain in three different tyrosine residues (985, 1,077, and 1,138). Tyrosine residue 1,138 anchors to STAT3, while residue 985 anchors to SHP2 and also to SOCS3, which negatively regulates leptin signaling (60). Through JAK2 activation, this long form of the leptin receptor is responsible for the main effects of leptin on the energy homeostasis and other neuroendocrine functions.

Leptin and LEPR are overexpressed in breast cancer as compared to non-cancer mammary epithelium (61). Moreover, immunohistochemical studies have shown overexpression of leptin receptors in breast cancer tissue samples of different stages, from primary to metastatic (62). In this sense, LEPR was also demonstrated to be necessary for maintaining CSC metastatic properties (63). This demonstrated that leptin can influence breast cancer cells not only by endocrine and/or paracrine actions, but also through autocrine pathways. High levels of leptin and overexpression of its receptors in obese women can lead to an increase in signaling, key in the development of breast cancer (29).

### Major Leptin Signaling Pathways in Breast Cancer

**Figure 3:**
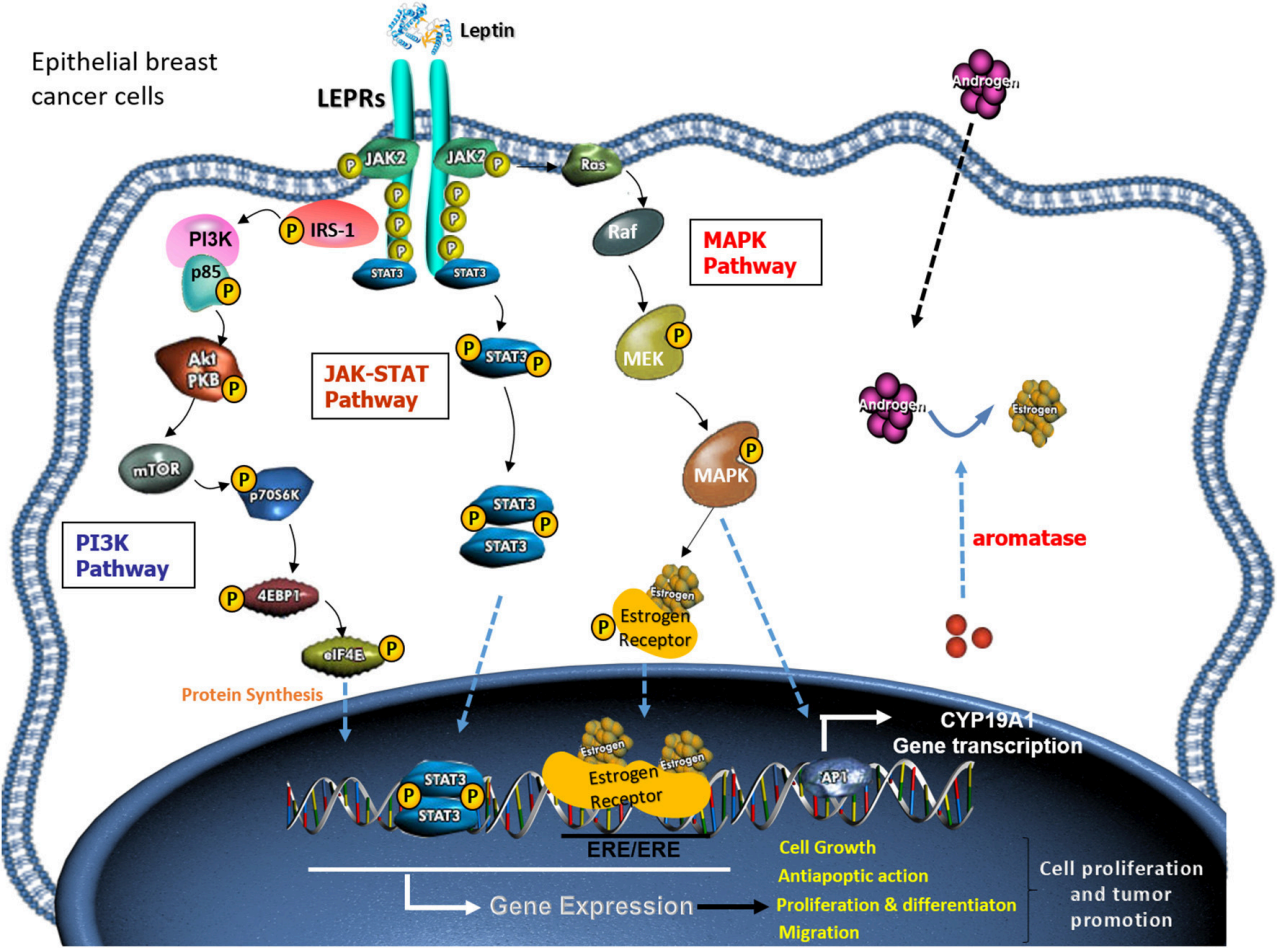
Mechanisms of leptin action in breast cancer cells. The cartoon shows the signaling pathways that mediate the leptin effects on breast cancer cells.

Through binding to its receptor, leptin stimulates a cascade of signaling events and elicit subsequent cellular effects (Figure 3). Leptin induces canonical (JAK2/STATs; MAPK/ERK 1/2, PI3K/AKT) and non-canonical signaling pathways (PKC, JNK, p38 MAPK, and AMPK) in diverse cellular types (64, 65).

Some multifunctional adaptor proteins and enzymes have been proposed to have a role participating in the activation of these signaling pathways. Despite the underlying mechanisms still remain poorly understood, proteins that have been previously related to cancer as well as to insulin signaling, such as APPL1 or Sam68, seem to positively mediate leptin induced phosphorylation of the main pathways activated by this hormone in breast cancer (66, 67).

#### JAK-STAT Pathway

The JAK/STAT signaling pathway participates in the regulation of proliferation, survival, mobility, and cellular apoptosis in different tissues and organs (68). It has been shown that leptin promotes the development and progression of breast cancer neoplastic cells by activating the JAK2/STAT3 pathway. After the binding of leptin to its receptor, activated JAK2 kinase phosphorylates the tyrosine 1,138 residue in the cytoplasmic domain of the leptin receptor. This provides an anchoring site for the STAT3 protein, which is recruited through its SH2 domain. Once STAT3 is bound to the receptor, it is phosphorylated by JAK2 and STAT3 proteins dimerize, thus allowing their translocation to the nucleus. There, they act as activators of the transcription of various genes, such as c-myc, cyclin D1, p21/waf1, c-jun, junB, erg-1, and Bcl-2, all of them involved in cell growth and proliferation (69). In addition, STAT3 dimers can activate the transcription of genes such as SOCS3, which modulate the effects of leptin on cells, as well as VEGF, involved in angiogenesis.

Signaling via JAK2/STAT3 is a critical regulatory component in carcinogenesis in breast cancer related to obesity (70). It has been demonstrated that leptin regulates the cell cycle and increases breast cancer cell growth by inducing cyclin D1 expression via STAT3 activation (38). A different proliferative effect of leptin in breast cancer cells involves the leptin mediated upregulation of human telomerase reverse transcriptase (hTERT) activity and expression in a dose-dependent fashion also involving STAT3 phosphorylation (71). Moreover, enhanced STAT3 signaling has been demonstrated to lead to altered expression in the key regulators of EMT to augment invasiveness and migration of mammary proliferating epithelial cells (72). Also, activated STAT3 seems to be crucial for the activation of CSC maintenance by leptin signaling in triple negative breast cancer (73). In this line, recent data from human breast-cancer-studies suggest that leptin-activated STAT3, also promotes cancer cell stemness and chemoresistance through the expression of critical enzymes for acid β-oxidation pathways (74).

#### MAPK Pathway

Phosphorylation on tyrosine 985 recruits SHP2 to the leptin receptor, contributing to the activation of the MAPK signaling pathway, which plays an important role in the activation of ERK 1/2, p38, and JNK (51). They also induce the activation of HIF-1α and NF-κB, and these transcription factors are linked to the regulation of VEGF/VEGFR-2 by leptin. In addition, MAPK induces the phosphorylation and activation of transcription factors, such as c-jun, c-fos, c-myc, and erg-1, which act in the nucleus by promoting expression of genes that regulate cell proliferation and differentiation. In addition, tyrosine 985 also is a binding site for SOCS3, playing an essential role in feedback inhibition of LEPR (75).

The role of leptin promoting growth via ERK pathway has been demonstrated in breast cancer models (76). In addition, this molecular pathway has been involved in the functional activation of the estrogen receptor alpha (ERα) (77, 78) as well as in the induction of aromatase mediated by leptin in breast tissue (79).

#### PI3K Pathway

Another protein with SH2 domain, SH2B1, also participates in LEPR signaling. Apart from increasing LEPR signaling through JAK2, SH2B1 can control specific signals on the insulin receptor substrate (IRS). IRS proteins control the PI3K pathway and the subsequent regulation of Akt signaling [Review in (80)]. Acting mainly through proteins phosphorylation, this pathway affects cellular properties more rapidly than other pathways.

PI3K pathway has been considered an important link between obesity, leptin and increased risk of breast cancer (31, 81). Specifically, it has been proposed that PI3K/AKT regulates leptin mediated-epithelial mesenchymal transition in breast cancer. In this process, this pathway has been suggested to participate in IL-8 (82) and pyruvate kinase M2 (PKM2) upregulation mediated by leptin (83). Furthermore, other recent study has proposed leptin to enhance the proliferation, migration and invasion of breast cancer cells via acetyl-CoA acetyltransferase 2 (ACAT2) up-regulation through the PI3K/AKT/SREBP2 signaling pathway (84).

### Leptin Crosstalk in Breast Cancer

Leptin has been linked to different stages and processes related to development and progression of breast cancer, involving the action of some mediators that participate in a more complex signaling transduction network. Thus, leptin functions are commonly reinforced through crosstalk with multiple oncogenes, cytokines and growth factors (60).

Leptin signaling enhances tumor formation, proliferation and invasion by activation of cancer stem cell signaling pathways. In this sense, leptin seems to participate in the expression of stem cell self-renewal transcription factors Nanog, SOX2, and OCT4 (63). Additionally, leptin induces canonical Wnt1 signal pathway functioning through β-catenin-dependent mechanisms. Several leptin dependent signaling kinases, including ERK-p90-RSK and Akt, which phosphorylate GSK3β, along with the increase of MTA1 expression by leptin, regulate the function of this Wnt pathway to promote epithelial-mesenchymal transition in breast cancer cells (85). In addition, leptin-Notch axis seems to play a role in breast cancer development, as it has been demonstrated in some models of breast cancer cells. A complex signaling crosstalk between leptin, Notch and interleukin-1 (IL-1) seems to be an important driver of leptin-induced oncogenic action (86).

Regarding angiogenesis regulation by leptin in breast cancer, some studies have shown that the activation of HIF-1α and NF-κB by leptin are essential in the regulation of VEGF, which promotes angiogenesis (87). In addition, leptin participates in the phosphorylation of VEGFR-2 (VEGF type 2 receptor) independently of VEGF in endothelial cells and breast cancer cells (60). Also the expression of this factor has been demonstrated to be increased through Notch, IL1, and leptin crosstalk (88), which could represent the integration of developmental, proinflammatory and pro-angiogenic signals.

In addition, various studies have integrated leptin and cAMP signaling pathways in breast cancer, specifically in triple negative breast cancer (89). Despite the role of cAMP and leptin, respectively, as growth suppressor and growth promoting factors in breast cancer cells, an antiproliferative interaction between leptin and cAMP elevation has been described (90). Thus, cAMP elevation seems to completely prevent leptin-induced migration of MDA-MB-231 breast cancer cells (91).

Other receptors have been proposed to be functionally important mediating the actions of leptin in breast cancer. Leptin and TGFβ1 seem to promote metastasis and stemness in breast cancer cells (92). As another example of this point of convergence with TGFβ signaling, leptin has been involved in metastasis and recurrence of breast cancer, likely participating in the inhibition of acetil CoA carboxylase 1 (ACC1) (93). In addition, the crosstalk between leptin and epidermal growth factor receptor (EGFR) signaling pathways could alter significantly the behavior of different cell types in cancer. Thus, it has been described bidirectional crosstalk between leptin and IGF-1 signaling, resulting in the activation of EGFR to promote proliferation and migration of triple negative breast cancer cells (39). An additional support for the existing link between leptin and other receptors is the close cooperation that was also demonstrated between leptin and HER2 (94). Not only HER2 seems to induce the expression of leptin in human breast epithelial cells (95), but also leptin can transactivate HER2 through the activation of the EGFR and the activation of JAK2, resulting in the growth of cancerous breast cells with overexpression of HER2 (14, 96). Therefore, the coexpression of HER2 and the leptin/LEPR system could contribute to increase the activity of HER2, reducing the sensitivity to monoclonal trastuzumab treatments for this type of breast cancer (29).

### Leptin and Estrogens

Estrogens play an important role in breast cancer, probably by stimulating cellular proliferation and oxidative metabolism mediated by various cytochrome P450 (CYP) enzymes such as CYP1A1 and CYP1B1. In breast tissues, the main estrogen, 17β-oestradiol (E2), is a ligand for the estrogen receptor α (ERα) and a substrate for the phase I enzyme cytochrome P450 CYP1B1. A large body of evidence has established the fundamental role of E2 and its receptor in the development and progression of breast cancer (97).

Leptin specifically enhances tumorigenicity of estrogen positive breast cancer cells (98). A bidirectional communication between LEPR and ERα was suggested by the statistically significant correlation between the expression of both receptors in breast cancer cells lines and ex *vivo* studies (99). Leptin has been suggested to induce CYP1B1 expression in ERα-positive breast cancer cells in a mechanism that involves AKT and ERK signaling pathways (78). Moreover, it has been demonstrated that leptin from adipose stromal/stem cells induces a differential pattern of gene expression in ERα-positive compared to negative breast cancer cells (100).

Leptin also exerts its effects by increasing cell viability and proliferation through crosstalk with estrogen receptor-alpha (ERα). Both STAT3 activation and ERK1/ERK2 signaling mediated by leptin have been described to act as a key event in ERα-dependent development of breast cancer (77, 101). Recently, a novel mechanism has been proposed for the role of leptin in breast cancer progression in ER-α positive cells. It involves JAK/STAT3-Akt signaling pathways in the suppression of the extracellular matrix protein CCN5, which acts as an anti-invasive element in cancer (102). Other studies have demonstrated that estrogen receptor signaling plays a key role in leptin-induced growth of breast cancer cells via autophagy activation (103).

An additional functional relation between leptin and estrogen signaling has been suggested based on the estrogen receptors status and the aromatase activity. In this sense, leptin promotes the synthesis of estrogen, which is related to an increased risk of breast cancer. Leptin was demonstrated to enhance aromatase expression in MCF7 cells (104) and this cytokine with some other inflammatory mediators are key stimulators of aromatase transcript expression also in adipose stromal breast cells (105).

Some mechanisms have been proposed for the effect of leptin on the aromatase expression. A positive association between leptin expression, LEPR, aromatase and MAPK and STAT3 activation has been suggested using tissue samples of patients with estrogen receptor positive breast cancer (106). Apart from that, PGE2 and leptin have been shown to drive aromatase expression via the suppression of the metabolic regulators LKB1/AMPK (107). Additionally, the increase in leptin-dependent aromatase expression has been correlated with COX-2 upregulation and cooperation among multiple signaling pathways (108). Specifically, a novel mechanism has been proposed based on leptin-dependent PKC/MAPK signaling, the suppression of p53, and HIF1α and PKM2 as direct mediators of aromatase expression (79).

### Leptin and Therapeutic Targets in Breast Cancer

Leptin network relationships have been correlated to prognosis and also pharmacological responses in some follow-up studies of breast cancer. For instance, patients with breast cancer who have high levels of leptin but with negative expression of estrogen hormone receptor have a higher survival rate. Therefore, a positive leptin/negative hormone receptor status is indicative of good response to chemotherapy and prognosis, whereas those with a positive leptin/positive hormone receptor profile have a poor response (22). Additionally, it has been demonstrated that leptin interferes with the action of tamoxifen under beta-estradiol stimulated conditions in ER-positive breast cancer cells (109, 110). Accounting for the role of obesity and leptin in breast cancer, several possible mechanisms have been suggested to potentially mediate drug resistance in tumor cells (111).

Different proposed therapeutic strategies for breast cancer treatment include the use of soluble leptin receptors, peptide-based leptin antagonists and leptin receptor blocking antibodies (45). Apart from this, events that promote leptin negative regulation are emerging as novel therapeutic targets for patients with breast cancer. The anti-leptin activity of 1,25(OH)2D3 in estrogen-sensitive tumors in women seems to mediate telomerase reverse transcriptase (hTERT) downregulation (112). Also, PPARγ ligands are demonstrated to inhibit leptin signaling mediated by MAPK/STAT3/Akt phosphorylation and counteract leptin stimulatory effect on estrogen signaling. Thus, PPARγ ligands have been suggested as therapeutic molecules for breast cancer treatment (113).

## The Paradox of the Benefits of Obesity in Cancer

On the other hand, even though the association between obesity, inflammation, and many different types of cancer is clear (114), some controversial data still remains, including breast cancer, where the dialog between the adipocyte and the breast cancer cells is well-known to potentiate not only the growth or invasion, but also treatment resistance (115). Thus, old and new data have also shown that moderately increased BMI improves the survival and response to therapy. However, this effect is lost when BMI increases to morbid obesity levels, leading to the concept of “obesity paradox” (116). This paradox is similar to that observed in other pathological conditions, such as cardiovascular disease (117). Although the obesity paradox in cancer is less studied, it has been related to the inadequate use of BMI for the overweight, since protective muscle mass may also contribute to BMI (118). Besides, weight depend on the stage of the disease, since cancer produces weight loss, and the time of measurement different studies may be misleading. Moreover, the excess of adipose tissue may represent an energy store that warrants further surviving time in some patients.

We think that both ideas may be conciliated, since chronic inflammation due to obesity may lead to cancer, whereas very low BMI may produce some immunodeficient state that favor the immune scape of cancer cells. On the other hand, once the cancer has been diagnosed, regardless the main cause of the tumor (tobacco, UV-radiation…), the proinflammatory state of the obese subject may be helpful for the immune response against the tumor. Finally, the increased fat stores may provide an energy reserve that may be useful for a longer survival time.

### The Beneficial Effect of Obesity in the Tumor Response to Immunotherapy

Immunotherapy has revolutionized cancer therapy in the last 10 years (119) and is now one of the cornerstone of both adjuvant and neoadjuvant therapies, alone or in combination with other chemotherapy or radiotherapy treatment (120, 121). In this line, immunotherapy has opened new horizons in breast cancer (122). Recent data has shown the beneficial effect of obesity in the efficacy of immunotherapy (123). Thus, cancer patients with overweight or obesity (BMI > 25) have shown to have a better response to treatment with anti-PD1/PD-L1 immune checkpoint inhibitors (124). This beneficial effect of increased BMI has been demonstrated in obese patients with metastatic melanoma (125, 126), where a mechanism mediated by chronic inflammation and increased C-reactive protein concentration has been proposed (127). Whether leptin contributes to this favorable effect of overweight on the response to immunotherapy warrants further investigation (128). Therefore, the association of obesity and the increased leptin levels with the response to treatment should be also investigated in breast cancer patients to better understand the response to immunotherapy as well as the better control of the appearance of side effects (Figure 2).

## Conclusions

Obesity is a known risk factor for the developing of breast cancer in post-menopausal women. The molecular mechanisms underlying the relationship between obesity and breast carcinogenesis involves estrogens, insulin, leptin, adiponectin, and inflammatory cytokines. Specifically, the activation of leptin signaling results in concurrent activation of multiple oncogenic pathways leading to an increased proliferation, epithelial-mesenchymal transition, migration and invasion of breast cancer cells. In this context, the knowledge of the complex molecular network of leptin signaling responsible for mammary carcinogenesis may provide novel ideas for the prevention and treatment of breast cancer associated to obesity. On the other hand, obesity has recently been found to be favorable for the response to immune checkpoint inhibitors in different tumors, even though this effect remains to be studied in breast cancer. Therefore, the role of leptin, and other adipokines on immunological response in cancer therapy should be considered in future studies.

## Author Contributions

All authors listed have made a substantial, direct and intellectual contribution to the work, and approved it for publication.

### Conflict of Interest Statement

The authors declare that the research was conducted in the absence of any commercial or financial relationships that could be construed as a potential conflict of interest.
